# Update on Biomarkers of Chronic Inflammatory Processes Underlying Diabetic Neuropathy

**DOI:** 10.3390/ijms251910395

**Published:** 2024-09-27

**Authors:** Adina Stoian, Carmen Muntean, Dragoș-Florin Babă, Andrei Manea, Lóránd Dénes, Zsuzsánna Simon-Szabó, Irina Bianca Kosovski, Enikő Nemes-Nagy, Florina Ioana Gliga, Mircea Stoian

**Affiliations:** 1 Department of Pathophysiology, George Emil Palade University of Medicine, Pharmacy, Science and Technology of Targu Mures, 540142 Targu Mures, Romania; adina.stoian@umfst.ro (A.S.); florina.gliga@umfst.ro (F.I.G.); 2 Department of Pediatrics 1, George Emil Palade University of Medicine, Pharmacy, Science and Technology of Targu Mures, 540142 Targu Mures, Romania; carmen.duicu@umfst.ro; 3 Emergency Institute for Cardiovascular Diseases and Transplantation, 540142 Targu Mures, Romania; dragos-florin.baba@umfst.ro; 4 Department of Cell and Molecular Biology, George Emil Palade University of Medicine, Pharmacy, Science and Technology of Targu Mures, 540142 Targu Mures, Romania; 5 Department of Radiology, Mureș County Emergency Hospital, 540136 Targu Mures, Romania; andrei17v1997@gmail.com; 6 Department of Anatomy and Embryology, Faculty of Medicine, George Emil Palade University of Medicine, Pharmacy, Science and Technology of Targu Mures, 540142 Targu Mures, Romania; 7 Department of Chemistry and Medical Biochemistry, Faculty of Medicine in English, George Emil Palade University of Medicine, Pharmacy, Science and Technology of Targu Mures, 540142 Targu Mures, Romania; eniko.nemes-nagy@umfst.ro; 8 Department of Anesthesiology and Intensive Care, George Emil Palade University of Medicine, Pharmacy, Sciences and Technology of Targu Mures, 540142 Targu Mures, Romania; mircea.stoian@umfst.ro

**Keywords:** diabetic neuropathy, type 2 diabetes mellitus (T2DM), inflammation, Toll-like receptor 4 (TLR4), caveolin 1, vitamin D

## Abstract

There is an increasing prevalence of diabetes mellitus (DM), particularly type 2 DM (T2DM), and its associated complications. T2DM is linked to insulin resistance, chronic inflammation, and oxidative stress, which can lead to both macrovascular and microvascular complications, including peripheral diabetic neuropathy (PDN). Inflammatory processes play a key role in the development and progression of T2DM and its complications, with specific markers like C-reactive protein (CRP), interleukins (ILs), and tumor necrosis factor (TNF)-α being associated with increased risk. Other key inflammatory markers such as nuclear factor kappa B (NF-κB) are activated under hyperglycemic and oxidative stress conditions and contribute to the aggravation of PDN by regulating inflammatory gene expression and enhancing endothelial dysfunction. Other important roles in the inflammatory processes are played by Toll-like receptors (TLRs), caveolin 1 (CAV1), and monocyte chemoattractant protein 1 (MCP1). There is a relationship between vitamin D deficiency and PDN, highlighting the critical role of vitamin D in regulating inflammation and immune responses. The involvement of macrophages in PDN is also suspected, emphasizing their role in chronic inflammation and nerve damage in diabetic patients. Vitamin D supplementation has been found to reduce neuropathy severity, decrease inflammatory markers, and improve glycemic control. These findings suggest that addressing vitamin D deficiency could offer therapeutic benefits for PDN. These molecular pathways are critical in understanding the pathogenesis of DM complications and may offer potential biomarkers or therapeutic targets including anti-inflammatory treatments, vitamin D supplementation, macrophage phenotype modulation, and lifestyle modifications, aimed at reducing inflammation and preventing PDN. Ongoing and more extensive clinical trials with the aim of investigating anti-inflammatory agents, TNF-α inhibitors, and antioxidants are needed to advance deeper into the understanding and treatment of painful diabetic neuropathy.

## 1. Introduction

### 1.1. Epidemiological Data on DM

The International Diabetes Federation estimated that over 536.6 million individuals aged 20–79 years were living with DM in 2021, predicting that this number will increase to over 783.2 million in 2045, affecting over 10% of the adult population [1]. DM, especially T2DM, has a prevalence that increases with age. Therefore, according to Saeedi et al., 19.9% of those aged 65–79 years develop DM [2]. For example, a DM prevalence of 12.8% has been reported in China [3].

Type 1 DM (T1DM) is an autoimmune disease characterized by the destruction of insulin-producing β cells in the pancreas. It is a chronic condition primarily mediated by T helper 1 (Th1) cells. The activation and infiltration of immune cells in the pancreatic islets leads to the gradual and subtle destruction of pancreatic β cells. Inflammatory cytokines such as IL-1, IL-6, TNF-α, and the interferon (IFN) family play crucial roles in the complex interactions between immune cells and pancreatic β cells, ultimately contributing to the development of T1DM [4].

Most cases (about 90%) of DM are T2DM, which is more common in individuals aged >45 years due to the increased prevalence of obesity, physical inactivity, and high-calorie diets. The prevalence of T2DM is also increasing in children and adolescents due to the same factors [5].

On the other hand, T2DM is a metabolic disease characterized by hyperglycemia, inadequate insulin release, insulin resistance, oxidative stress, and chronic inflammation, which can affect multiple organs and tissues [6]. Genetic and environmental factors in T2DM lead to early cell death in pancreatic islets and adipose tissue, which triggers the release of pro-inflammatory chemokines and cytokines. They attract immune cells (macrophages, neutrophils, dendritic cells, natural killer (NK) cells, and T cells) to infiltrate the pancreatic islets and adipose tissue, initiating an immune attack. The damage to β cells results in decreased insulin secretion capacity. These inflammatory cells release more cytokines, accelerating β cell death [4].

### 1.2. Role of Inflammation in DM

Inflammation is the body’s defense process in which the immune system recognizes and attempts to eliminate foreign and harmful stimuli, initiating the healing process [7]. Chronic inflammation is a persistent and prolonged process that can last for months or years, unlike acute inflammation, which is a quick and short-lived response. Its continued presence may result from prolonged tissue injury and immune response, with the body’s inability to eliminate the causative factor [8]. It can be caused by infectious agents, chronic exposure to irritating factors, autoimmune phenomena, defects in cell-mediated inflammation, oxidative stress, mitochondrial dysfunction, free radical formation, and advanced glycation end products (AGEs) [7]. Chronic inflammation is characterized by the infiltration of tissues by macrophages, lymphocytes, and plasma cells which release inflammatory cytokines such as IL-1 and TNF-α, as well as growth factors and enzymes that sustain the inflammatory process. This perpetuation occurs through the recruitment of new leukocytes via chemotaxis and diapedesis. The ongoing inflammatory response leads to secondary tissue damage and activates reparative mechanisms, causing fibrosis and granulomas, altering normal functionality [7].

Inflammatory processes occurring in adipose tissue, the pancreas islets, muscles, and the liver may precede the features of DM becoming evident [9,10]. Monocytes and neutrophils from the blood and adipose tissue are the primary sources of inflammatory cytokines. The gene expression and phenotypes of neutrophils and monocytes can be altered under chronic high blood sugar levels [11]. Low-grade inflammation, marked by the release of pro-inflammatory cytokines, contributes to the development of T2DM [12]. Inflammation appears more aggressive in T2DM than in T1DM [13]. In addition, the expression of IL-1, IL-6, IL-8, TNF-α, intercellular adhesion molecule 1 (ICAM1), and cyclooxygenase 2 (COX2) was higher in monocytes from patients with T2DM than from controls [13,14]. Inflammation and leukocytosis further increase insulin resistance, a characteristic of T2DM. Obesity, which is common in patients with T2DM, induces the dysregulation of adipokines and some transcription factors, with chronic tissue inflammation and secondary insulin resistance in these tissues [14,15]. Inflammatory syndrome itself is considered a risk factor for developing DM. Elevated levels of CRP, interleukin (IL)-1, IL-6, C-X-C motif chemokine ligand 10 (CXCL10), tumor necrosis factor (TNF)-α, IL-12 (p70), IL-13, and IL-17A are associated with an increased risk of T2DM and diabetic neuropathy [6,11]. In addition, chronic systemic inflammation is recognized as part of insulin resistance syndrome, T2DM, and obesity and is characterized by increased IL-6 and CRP levels, as well as decreased levels of adiponectin (ADIPOQ) and IL-10 [16].

Additionally, excess glucose can interfere with various metabolic pathways, causing inflammatory nerve damage [17]. A significant percentage of these individuals (about 50%) will develop diabetic neuropathy [2]. Inflammation has also been used as a biomarker in predicting the development of microvascular and macrovascular complications of DM [18].

### 1.3. The Importance of Studying the Inflammatory Process in PDN

T2DM is associated with both macrovascular complications (e.g., cerebrovascular disease, coronary artery disease, and peripheral artery disease) and microvascular complications (e.g., diabetic retinopathy, diabetic nephropathy, and diabetic neuropathy) [10,19,20].

Predictions suggest that by 2045, around 700 million individuals will be diagnosed with DM [21]. This increase will also lead to increases in associated complications, individual discomfort, and the financial burden on society. The incidence of complications increases with DM duration and age, often making it difficult to distinguish between peripheral nerve changes due to aging or DM-induced dysfunctions [22]. Neuropathy and atherosclerosis in the arteries of the lower limbs and changes in the bone anatomy of the leg predispose one to the development of ulcers, a complication associated with many hospitalizations and the exacerbation of inflammatory processes [23]. The wound-healing process is influenced by the levels of pro-inflammatory and anti-inflammatory cytokines. The disturbance of the immune system with increases in inflammatory cytokines IL-6 and TNF-α can adversely affect this process, with the appearance of diabetic foot [24]. The consequences can be severe, including the development of foot ulcers and even amputation [2].

PDN encompasses a variety of symptoms suggestive of sensory and motor dysfunctions of the peripheral nervous system. In PDN, there is a change in superficial sensitivity, often accompanied by hyperalgesia and allodynia, with a stocking–glove pattern and with progression, sometimes even leading to anesthesia [25]. PDN can affect both myelinated and unmyelinated peripheral nerve fibers, and it is one of the most common chronic complications of DM. Pain usually occurs secondary to the impairment of thin, unmyelinated fibers. In contrast, the impairment of large-diameter myelinated nerve fibers may not cause pain but can lead to gait instability, increasing the risk of falls and injury [14]. In T1DM, PDN typically becomes evident after several years of prolonged hyperglycemia, whereas in T2DM, PDN can be present from the onset of the disease or may become evident after a short period of poor glycemic control [26]. The American Diabetes Association (ADA) recommends neurological screening for patients with T2DM at the time of diagnosis and at 5 years from the onset of T1DM [27].

Diabetic autonomic neuropathy (DAN) is a commonly underestimated complication of DM that affects the autonomic nervous system circuitry, leading to a wide spectrum of cardiovascular, gastrointestinal, genitourinary, sudomotor, and autonomic pupillary manifestations. Subclinical manifestations of DAN may also appear early, within the first year of T2DM diagnosis and approximately 2 years after the onset of T1DM [28]. The subclinical manifestations include mild or nonspecific symptoms that become evident only through specific tests. Patients may not recognize these symptoms, as they do not significantly interfere with daily life, such as slight changes in heart rate variability or mild gastrointestinal symptoms [29]. However, clinical manifestations become evident only after several years of disease progression [28].

Diabetic neuropathy results from a complex interaction between hyperglycemia, dyslipidemia, and insulin resistance. Hyperglycemia activates multiple biological pathways, such as the polyol, glycolysis, hexosamine, and advanced glycation end product pathways, leading to oxidative stress, mitochondrial damage, and inflammation. These processes result in endoplasmic reticulum stress, deoxyribonucleic acid (DNA) damage, and an increased inflammatory response. Persistent mild inflammation, caused by ongoing stress or dysfunction, and increased levels of glucose, lipoproteins, and oxidative and glycated proteins altogether contribute to nerve damage [4].

### 1.4. The Purpose and Significance of This Review

The mechanisms underlying abnormal nociceptive sensations in DM are unclear. While multiple mechanisms are being investigated, neuroinflammation has been recognized as an important factor in its etiopathogenesis [2,6]. The purpose of this review is to provide an update on chronic inflammatory processes, key inflammatory markers, and potential protective factors related to the development of PDN in DM, particularly T2DM. The review also covers preventive measures and therapeutic alternatives.

## 2. Toll-like Receptors (TLRs)

**Figure 1:**
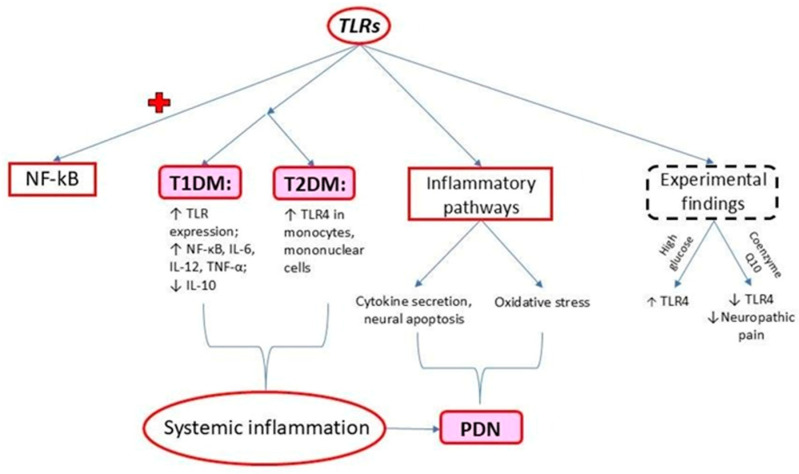
Main effects produced by increased TLR expression in DM; + activation.

TLRs recognize pathogen-associated molecules and molecules associated with endogenous damage, activating NF-κB. A general overview of the effect of TLRs is presented in Figure 1, with further details provided in this section.

Increased TLR expression in mice with T1DM without associated obesity was accompanied by increased NF-κB, IL-6, IL-12, and TNF-α levels and decreased anti-inflammatory cytokine IL-10 levels [14,30].

TLR4 expression in mononuclear cells was fivefold higher in patients with T2DM than nondiabetic controls, suggesting that TLR-induced signaling could be involved in DM development [14,31]. Other authors like Dasu et al. suggest that TLR4 expression is induced by high glucose in cell culture experimental studies [32]. TLRs can influence the secretion of cytokines and chemokines involved in early sensitive neuropathy, affecting axonal growth and inducing the apoptosis of dorsal root ganglion (DRG) neurons under hyperglycemic conditions [33].

Experimental evidence suggests that the antioxidant coenzyme Q10 (CoQ10), a systemic free radical scavenger, suppresses neuropathic pain in T2DM by downregulating TLR4 expression. Coenzyme Q10, also known as ubiquinone, is a fat-soluble molecule in cell membranes. It plays a crucial role in transferring electrons within the mitochondrial respiratory chain and in producing adenosine triphosphate (ATP). CoQ10 stimulates the production of antioxidants such as superoxide dismutase to mitigate oxidative stress, lowers lipid peroxidation, and improves blood flow by preserving nitric oxide (NO) [34]. Zhang et al. found that TLR4 expression in monocytes was higher in patients with T2DM and PDN than in patients with T2DM without PDN. Previous studies have shown that the expression and activation of TLR4 in human monocytes are higher under hyperglycemic conditions, potentially leading to exacerbated tissue inflammation [35]. These results suggest a link between increased TLR4 expression in monocytes, systemic inflammation, and PDN [35,36]. The increased TLR4 expression in patients with PDN suggests that inflammation levels are higher than those in patients with T2DM without PDN [36]. Zhu et al. showed that the significant upregulation of TLR4 in monocytes might contribute to inflammation in PDN [36].

Inducing factors of inflammation cause oxidative stress and mitochondrial dysfunction with the increased production of free radical molecules, oxidized lipoproteins, and AGEs. The released mitochondrial proteins, including cytochrome C (Cyt C), modulate apoptosis. The inflammatory pathways lead to the development of insulin resistance through inhibiting signaling downstream of the insulin receptor [37,38].

Schwann cells cultured under high-glucose conditions showed increased apoptosis and TLR4 overexpression (receptors activated by lipopolysaccharides) and increased TNF-α production [39]. Increased TLR4 messenger ribonucleic acid (mRNA) and TNF-α levels were observed in the spinal cords of rats with streptozocin (STZ)-induced DM and correlated positively with mechanical and thermal hypersensitivity, supporting their role in the development of diabetic neuropathy [40]. Yan et al. reported that TLR4 expression was gradually upregulated in the spinal cords of rats with STZ-induced DM and PDN and correlated positively with the expression of pro-inflammatory cytokines [40].

Another study involving 246 patients with T1DM and 530 patients with T2DM reported that PDN in T1DM was not associated with a single-nucleotide polymorphism in the TLR4 gene. However, the TLR4 gene was involved in the appearance of PDN in patients with T2DM [41].

The increase in TLR4 expression was reported to be positively correlated with plasma concentrations of TNF-α and IL-6. It might also be a response associated with an inflammatory state and the occurrence of PDN, and might not mandatorily be a trigger [36].

## 3. Caveolin 1 (CAV1)

**Figure 2:**
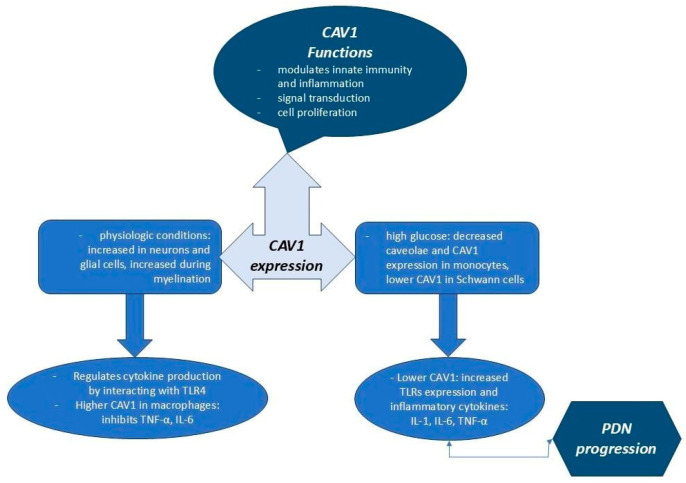
CAV1 functionality in physiologic and pathologic conditions.

CAV1 is a 22 kDa membrane protein with multiple roles, including regulating cholesterol homeostasis, cell proliferation, signal transduction, and receptor-independent endocytosis. It also modulates innate immunity and inflammation. A general overview of the CAV1 functions and effects in physiologic and pathologic conditions (prolonged hyperglycemia) is presented in Figure 2, with further details provided in this section.

The effects of decreased CAV1 expression on the production of pro-inflammatory cytokines such as IL-1, IL-6, and TNF-α could be mediated by its interaction with TLR4 and the regulation of TLR4 activation. CAV1 regulates receptor signaling by directly binding to receptors of various molecules [36].

Caveolin-1 (CAV1) is expressed in neurons and glial cells of the central nervous system (CNS). CAV1 expression increases during the myelination process, where it plays a regulatory role in cell signaling in glial cells. Chronic hyperglycemia is associated with a decrease in CAV1 expression in Schwann cells. In the context of painful PDN, CAV1 appears to play a regulatory role. Diabetic mice lacking CAV1 show greater deficits in motor nerve conduction and sensitivity to thermal and mechanical stimuli, suggesting that CAV1 may help attenuate PDN progression [42]. New research indicates that CAV1 may have a role in reducing inflammation. Studies have shown that increasing CAV1 in the macrophages of murine models inhibited the production of TNF-α and IL-6 [43]. Additionally, CAV1 is thought to exert anti-inflammatory effects by binding to TLR4. Monocytes, in particular, seem sensitive to changes in CAV1 levels, with lower CAV1 expression than other cell types, suggesting that the interaction between CAV1 and TLR4 in monocytes may be involved in the inflammatory process of PDN in T2DM [36,44]. A study involving male Sprague Dawley rats with T2DM induced by a high-fat, high-sugar diet for eight weeks followed by an intraperitoneal injection of a low STZ dose showed that CAV1 was persistently upregulated in the spinal cords of rats with diabetic neuropathic damage, concluding that it plays an important role in peripheral neuropathic pain [45].

In previous studies, Hayashi et al. found that higher glucose levels are associated with the decreased number and size of caveolae and reduced CAV1 expression in monocytes. Other studies also noted that decreased CAV1 expression associated with high glucose levels was connected to PDN progression [46,47]. Additionally, Zhu et al. demonstrated that the lower CAV1 expression in the monocytes of patients with T2DM and PDN than patients with T2DM but without PDN correlated negatively with TLR4 expression and the levels of pro-inflammatory cytokines (e.g., IL-6 and TNF-α), suggesting that reduced CAV1 could be a predictive marker for PDN [36].

## 4. NF-κB

NF-κB is a transcription factor that regulates the expression of genes involved in controlling biological processes in the CNS, such as synaptic plasticity and neurogenesis. NF-κB is found in the cytoplasm in an inactive form under the inhibition of regulatory proteins known as inhibitors of κB (IκB) [6,48]. Phosphorylation of the IκB inhibitory proteins by various signals allows the NF-κB complex to translocate into the nucleus, where it binds to DNA and affects the expression of various genes. This NF-κB signaling pathway is associated with some models of inflammatory painful neuropathy, supporting its role in the development of nociceptive disorders in experimental DM [49].

**Figure 3:**
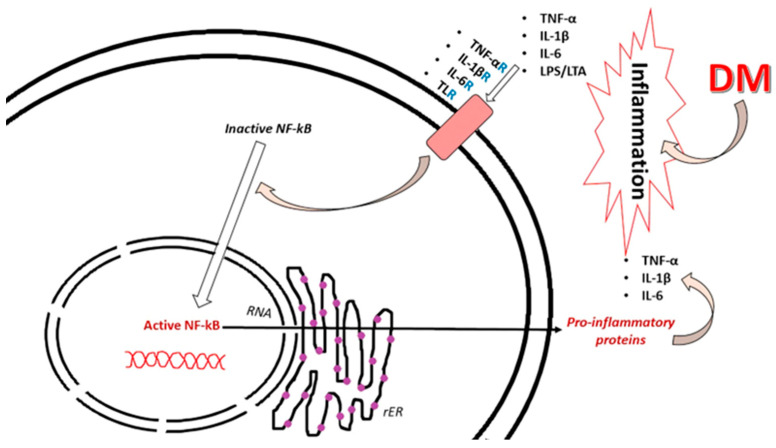
The pro-inflammatory mechanism involved in the presence of DM. The inflammatory process from DM, involving the secretion of cytokines, such as TNF-α, IL-1β, and IL-6 acting on its receptors (TNF-αR, IL-1βR, and IL-6R), together with the activation of TLR made by lipopolysaccharides (LPSs) and lipoteichoic acid (LTA), conduces to the activation path of NF-κB. The activation of NF-κB further stimulates the translation and transcription process, with the rough endoplasmic reticulum (rER) contribution generating pro-inflammatory proteins that create a vicious cycle.

NF-κB can be activated by stimuli such as hyperglycemia, pro-inflammatory cytokines (e.g., TNF-α and IL-6), and oxidative stress [50]. It is released during cell damage and inflammation and is involved in the stress response, adaptive immunity, and B cell development [51]. Studies have shown that NF-κB influences the activation, differentiation, and survival of T cells and other cells of the innate immune system and, in turn, is involved in the production of pro-inflammatory cytokines by modulating immunological responses, as represented in Figure 3.

NF-κB is expressed by endothelial cells, correlating with their dysfunction, and its overexpression damages them and is associated with diabetic neuropathy progression [52,53,54].

Activation of the NF-κB axis in experimental studies regulates the expression of some inflammatory genes such as COX-2, lipoxygenase, NO-synthase, and endothelin-1 and triggers a series of immune and inflammatory responses that can cause cell damage and the increased expression of adhesion molecules [50,55]. Thus, ischemic reperfusion and injury in an experimental model of STZ-induced DM led to the overproduction of NF-κB from endothelial cells and Schwann cells from the sciatic nerve followed by the increased expression of ICAM-1 with the excessive infiltration of monocytes and macrophages, suggesting that NF-κB has a role in mediating the increased inflammatory response at the nerve level in DM [56].

Nuclear factor erythroid 2 related factor-2 (Nrf2) is an important transcription factor in regulating the body’s antioxidant defense system. It does this by increasing the production of detoxifying enzymes and enhancing the antioxidant capacity. The activity of Nrf2 and NF-κB is coordinated to maintain redox homeostasis in healthy cells [57]. In PDN, this regulation is disturbed. Li et al. showed that mice deficient in Nrf2 exhibit increased expression of genes encoding inflammatory mediators, including interleukins, TNF-α, inducible (i)NOS, and COX-2. They highlighted that Nrf2 deficiency is linked to pro-inflammatory responses mediated by NF-κB [58].

## 5. Immune-Related Events

### 5.1. Immune Cells

Monocytes and macrophages are front-line cells in the body’s immune system. They rapidly accumulate at the site of an injury [18]. Monocytes differentiate into macrophages, and depending on the stimuli, they can differentiate into M1 (pro-inflammatory) macrophages under increased IFN-γ and TNF-α levels [59,60,61] or M2 (anti-inflammatory) macrophages under increased IL-4 and IL-13 levels [62]. In turn, M1 macrophages secrete pro-inflammatory cytokines that can increase insulin resistance [63], and M2 macrophages secrete anti-inflammatory cytokines that contribute to tissue repair and healing [64].

**Figure 4:**
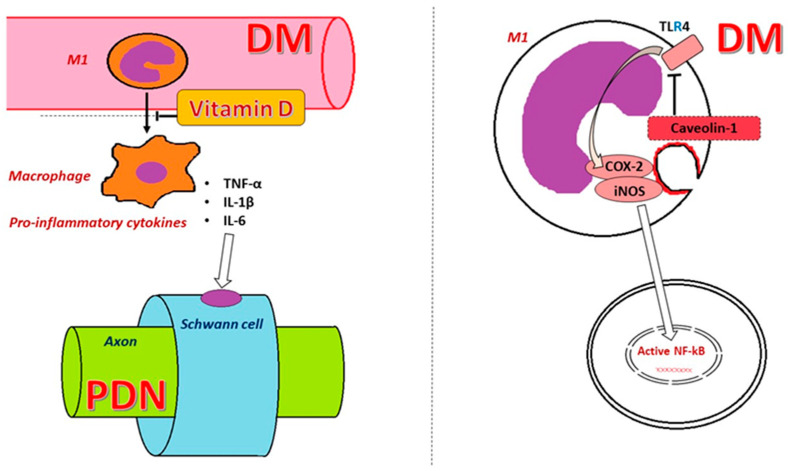
Cellular mechanism of DM in the development of PDN. In DM, the overstimulation of M1 TLR4 results in the release of iNOS and the activation of the COX-2 pathway. COX-2 stimulation and iNOS further activate NF-κB, leading to pro-inflammatory status. The secretion of TNF-α, IL-1β, and IL-6 has an impact on Schwann cells, causing PDN. Caveolin-1, a cell membrane protein, has an inhibitory role against the activation of M1 TLR4, showing promising preliminary results in DM.

Immune cells play a primary role in the inflammatory response. Their long-term stimulation under chronic hyperglycemic conditions leads to their activation, followed by a series of responses. Macrophage activation determines the initiation of signaling pathways with the release of TNF-α, IL-1β, IL-6, or other inflammatory signaling factors such as NF-κB, resulting in organ damage [18] (Figure 4).

DM is associated with persistent inflammation accompanied by a pro-inflammatory phenotype at the level of monocytes/macrophages [65]. This systemic inflammatory response may result from a dysfunctional pro-inflammatory M1 macrophage phenotype, contributing to the etiopathogenesis of diabetic neuropathy, especially its painful forms. The transition from the pro-inflammatory M1 phenotype to the anti-inflammatory M2 phenotype of macrophages leads to the remission of inflammation. However, in patients with DM, macrophages constantly show an M1 phenotype, producing pro-inflammatory cytokines and proteases, promoting oxidative stress with myelin degradation and blocking nerve regeneration [66,67]. M1 macrophages also induce changes in the pancreatic cells, aggravating DM through hyperglycemia [68].

The in vitro cultivation of macrophages in a high-glucose environment induced a dysfunctional phenotype after a pro-inflammatory challenge. In addition, the effects imprinted in the phenotypes of monocytes after prolonged exposure to hyperglycemia seemed to persist even after the differentiation of macrophages under normal glucose levels. These phenotypic changes are possibly irreversible, suggesting that patients with T2DM and controlled glycemia remain prone to developing cardiovascular complications or diabetic neuropathy due to a pro-inflammatory monocyte/macrophage phenotype [63].

Macrophages can affect the peripheral nervous tissue, where they regulate neuroinflammation and seem to play a role in the development of PDN. Analyzing the sciatic nerves from mice with PDN revealed elevated pro-inflammatory markers of M1 macrophages, such as TNF-α and IL-1β, and reduced anti-inflammatory markers of M2 macrophages, such as IL-10 and transforming growth factor (TGF)-β, and the interference and polarization of macrophages toward the M2 phenotype positively affected the improvement in DM complications [69]. In mice with STZ-induced T1DM and ob/ob obese mice with T2DM and leptin (LEP) deficiency, a slight infiltration of the sciatic nerve by macrophages and T cells was observed, which correlated with the loss of both myelinated and unmyelinated nerve fibers [70,71]. Studies on animals with T1DM induced by STZ have also revealed T cell and neutrophil infiltration in the dorsal root ganglia, particularly in the later stages of the disease [72].The role of macrophages in the microvascular complications of DM has been documented by previous studies that showed their accumulation in the case of renal dysfunction, M1 macrophages damaging the integrity and prompting the apoptosis of podocytes in rats with diabetic nephropathy, and M2 macrophages exerting protective effects in the kidneys [18,73,74]. While the activation of both M1 and M2 macrophages was observed in diabetic retinopathy, as it progressed, the number of M2 macrophages decreased in parallel with retinal dysfunction [75]. Exposing human macrophages to a hyperglycemic environment in vitro changed their inflammatory response [76]. These cells represent the final effectors in various pathological conditions associated with DM, such as ulcers or peripheral nerve injuries [65]. The cluster of differentiation CD163 molecule (CD163) is a cell surface receptor found on circulating monocytes and tissue macrophages [77]. Macrophages expressing CD163 are found especially in the resolution phase of acute inflammation and healing but also in chronic inflammation [65,78]. The number of CD163+ macrophages was observed to be reduced in patients with DM [65,79]. Patients with T1DM who experience neuropathic pain show an increase in CD4+ memory T cells, alongside elevated monocyte levels in the peripheral blood [72].

Alvarado-Vázquez et al. highlighted that macrophages from patients with T2DM without PDN showed increased IL-6 production without significant changes in TNF-α, MCP1, or anti-inflammatory cytokine production [65]. These observations suggest that T2DM can influence the monocyte/macrophage phenotype, and correcting these dysfunctional macrophages’ phenotypes could benefit patients with T2DM, even those with controlled glycemia [52]. The macrophages of patients with PDN show a dysfunction characterized by the reduced production of the pro-inflammatory chemokine MCP1 and the anti-inflammatory cytokine IL-10, which play a role in resolving the inflammatory process [65,80,81]. MCP1 is a potent chemoattractant for macrophages. The inadequate response to deficient chemoattractants and insufficient signaling for the anti-inflammatory M2 phenotype induced by IL-10 explain the increased risk of chronic leg ulcers [82]. Sural nerve biopsies were taken from patients with PDN to evaluate the types of immune cells involved. They revealed more CD3+ T cells in the nerves of patients with DM, a lack of membrane-spanning 4-domains A1 (MS4A1/CD20)+ B cells, and an excess of CD68 molecule (CD68)+ macrophages, with increased reactivity in the peripheral nerves to TNF-α and IL-6 receptors, and to a lesser extent to IL-1β and IL-1α [22,83]. A cytotoxic effect on Schwann cells exerted by CD8+ lymphocytes is also suspected [84].

Activated microglia in hyperglycemic conditions can release neuroactive and neuromodulatory factors such as pro-inflammatory cytokines, prostaglandins, NO, and reactive oxygen species. These factors seems to be involved in hyperalgesia and pain in patients with PDN [84,85].

### 5.2. Cytokines and Chemokines

**Figure 5:**
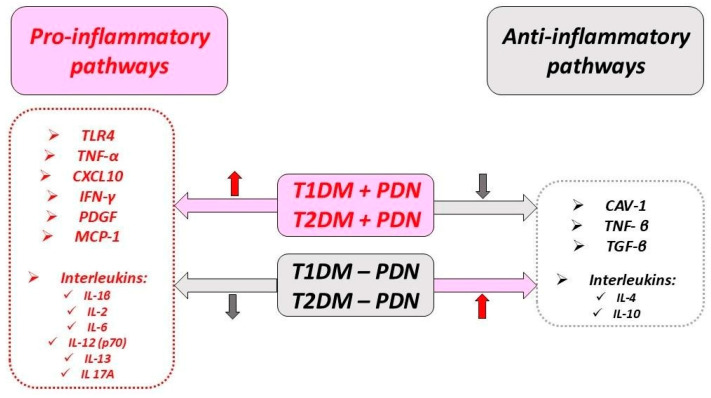
Pro-inflammatory and anti-inflammatory pathways and their relationship with the occurrence of PDN.

Cytokines and chemokines are small molecular proteins that play a role in both cellular pathological processes and their interactions, promoting DM complications [18]. A general overview of the inflammatory and anti-inflammatory pathways in DM and associated PDN is presented in Figure 5, with further details provided in this section.

Blood monocytes and tissue macrophages produce ILs, which are essential for the proper functioning of the immune system. In infections, ILs released by immune cells act on the surface of the target cells, causing a change in their behavior and activation [86,87].

Giving diabetic rats fish oil supplements helped improve their general condition. Li et al. showed that in diabetic rats, mechanical allodynia and thermal hyperalgesia were accompanied by increased TNF-α levels in the DRG. Administering fish oil attenuated the pain in diabetic rats and was followed by a decrease in TNF-α levels, suggesting that reducing this inflammatory cytokine could ameliorate painful diabetic neuropathy [6].

Hinder et al. examined gene expression in db/db diabetic mice before and after PDN development and in control mice without DM. They found higher levels of IFN-γ, IL-10, and matrix metallopeptidase 12 (MMP12) in the sciatic nerves of diabetic mice than in nondiabetic mice [88]. Other studies have reported LEP deficiency and upregulation of the NF-κB and Janus kinase/signal transducer and activator of transcription (JAK/STAT) pathways, along with the dysregulation of IL-2, IL-6, and IL-10 cytokines in diabetic mouse models [89]. Additionally, Yanik et al. indicated that IL-10 may alleviate pain in animal models with painful PDN [90]. Inhibiting TLR4 signaling in the spinal cords of diabetic rats attenuated mechanical hyperalgesia and decreased TNF-α levels. The continuous delivery of IL-10 to the nerve fibers in the DRG blocked the nociceptive response and decreased TLR4 expression in experimental animals with induced DM [90,91,92].

Various studies have investigated the levels of inflammatory cytokines in patients with PDN, finding elevated levels of CRP, LEP, TNF-α, fibrinogen, and IL-6 [93,94]. Other studies have demonstrated that inflammatory cytokines are involved in the development of neuropathic pain. NF-κB promotes the transcription of TNF-α and IL-1β, which in turn activate NF-κB [6].

TNF-α is produced especially by monocytes, macrophages, and T lymphocytes and functions as a signaling protein, influencing various molecular and cellular processes, including cell migration, adhesion, angiogenesis, and apoptosis. TNF-α activates T lymphocytes and macrophages, inducing the production of other cytokines and cell adhesion molecules and potentially inducing insulin resistance by reducing insulin signaling and promoting the serine phosphorylation of insulin receptor substrate 1 (IRS1) [86,91]. In DM, TNF-α can also be produced by Schwann cells and endoneurial macrophages [95]. Patients with PDN had increased serum levels of both TNF-α and its soluble receptors [96]. TNF-α has been implicated in the pathogenesis of insulin resistance and the initiation of the development of T2DM [97]. Patients with painful PDN were found to have increased expression levels of TNF-α in macrophages [98]. TNF-α reduces the nerve conduction velocity by damaging lamellar structures and peripheral axons [18,99]. Rosane et al. found that TNF-α can increase the apoptosis of Schwann cells, resulting in peripheral nerve damage [100,101]. TNF-α affects sensitive neurons and damages sensitive peripheral nerve fibers, resulting in pain [102]. Physical exercise has been shown to improve and reduce TNF-α and CRP levels, leading to better balance and reduced sensitivity symptoms [103]. One study evaluating patients with T1DM with and without PDN found that those with PDN had higher prescribed doses of insulin, hemoglobin A1c (HbA1c) levels, and TNF receptor superfamily member 1 (TNFRSF1A) and 2 (TNFRSF1B) levels [104]. Another study demonstrated elevated serum TNF-α levels and reduced serum IL-10 levels in patients with prediabetes and patients with T2DM and PDN compared with controls without DM [102]. Duksal et al. examined 144 subjects—50 patients with prediabetes, 50 patients with T2DM, and 44 controls—and reported elevated plasma levels of TNF-α in patients with T2DM compared to the controls and lower levels of IL-10 in patients with T2DM and prediabetes compared to the controls but did not find correlations between these biomarkers and conduction in distal sensory nerves (sural, dorsal plantar, and medial plantar) [105]. Other clinical and experimental studies have reported associations between TNF-α and conduction velocities in sensory nerves, adjusted for age, in patients with T2DM [104]. Similarly, Hussain et al. reported higher levels of TNF-α in patients with PDN than without PDN [106].

An increase in serum TNF-α levels has been observed in patients with T2DM [6]. Zeng et al. examined 55 patients with prediabetes, 55 patients with T2DM, and 48 controls, reporting significantly higher TNF-α levels in patients with T2DM than in patients with prediabetes and controls. However, the increase in TNF-α levels in patients with prediabetes compared to controls was not statistically significant. Spearman’s rank correlation coefficient (ρ) indicated a positive correlation between HbA1c and TNF-α levels (ρ = 0.411, *p* < 0.001). TNF-α levels correlated positively with Neuropathy Disability Scores (NDSs; ρ = 0.796, *p* < 0.001) and Neuropathy Symptom Scores (NSSs; ρ = 0.489, *p* < 0.001). In addition, NDSs correlated negatively with the sensory nerve action potential (SNAP) amplitude of the dorsal sural nerve (ρ = −0.326, *p* < 0.05) and the medial plantar nerve (ρ = −0.402, *p* < 0.05), as well as the nerve conduction velocity of the dorsal sural nerve (ρ = −0.431, *p* < 0.05). However, plasma levels of TNF-α, IL-10, and HbA1c did not correlate with the SNAP amplitude of the examined nerves or with nerve conduction velocity [12].

Alvarado-Vázquez et al. showed that increased IL-6 levels correlated positively with insulin resistance in patients with obesity [65]. Moreover, cardiovascular complications and atherosclerotic disease were frequently associated with DM and obesity, and IL-6 and TNF-α levels were elevated in human atheromatous lesions [107]. Vascular changes reduce the supply of oxygen and nutrients to the peripheral nerve fibers, favoring the development of PDN [65]. IL-6 favors the expansion of ulcers in the lower limbs in diabetic foot [108]. Blüher et al. examined 142 Caucasian men and women, including patients without T2DM, patients with impaired glucose tolerance (IGT), and patients with T2DM. They found that obesity, IGT, and T2DM were associated with increased IL-6 and CRP levels [16]. Consistent with other studies [109,110], they also found that IGT and T2DM were associated with decreased IL-10 and ADIPOQ levels [16]. Additionally, Magrinelli et al. highlighted increased serum IL-6 levels in over 40% of patients with DM, which correlated negatively with the function of peripheral myelinated and non-myelinated nerve fibers and were associated with neuropathic pain [111]. Bäckryd et al. showed that patients with neuropathic pain had higher IL-6 levels [112], and Angst et al. reported increased IL-6 levels in patients with painful PDN [113]. Other data suggest that IL-6 affects glial cells and neurons and is associated with the development of PDN [114].

However, the data are somewhat contradictory, and there are also experimental studies that reported that treating diabetic rats with pharmacological doses of IL-6 improved sensory and motor nerve conduction velocity, improved tactile allodynia, and altered thermal nociception by promoting increased nerve blood flow. Further studies in humans are needed to clarify the role of IL-6 [115,116,117].

The data reported on the association between IL-10 and T2DM are contradictory. Some studies have reported lower IL-10 levels in patients with IGT and patients with T2DM than in controls [16]. Increased IL-10 levels were associated with a lower prevalence of T2DM and metabolic syndrome and lower HbA1c levels [118]. In contrast, Pham et al. reported higher IL-10 levels in patients with T2DM compared to healthy controls [92]. Zeng et al. reported that anti-inflammatory IL-10 levels were significantly lower in patients with prediabetes than in controls and in patients with T2DM than in patients with prediabetes. lL-10 levels correlated negatively with NDSs (ρ = −0.662, *p* < 0.001) and NSSs (ρ = −0.422, *p* < 0.001). In addition, IL-10 levels correlated negatively with TNF-α levels (ρ = 0.483, *p* < 0.001) and HbA1c levels (ρ = −0.514, *p* < 0.001) [12].

Studies have shown that the activation of inflammatory cascades is important in the onset and persistence of neuropathic pain. Patients with painful diabetic neuropathy were found to have increased mRNA and blood protein levels of IL-1β, IL-2, TNF-α, CRP, and LEP, which could be associated with neuronal dysfunction [94,119]. Xiaohua et al. reported that inflammatory cytokines, such as IL-2, IL-6, IL-10, TNF-α, IFN-γ, and CRP, play significant roles in painful PDN development and progression. They revealed that patients with painful PDN had significantly higher levels of IL-6 and TNF-α than those with painless PDN and those with T2DM without PDN (*p* < 0.01) [120].

CRP is a plasma protein produced by the liver whose levels increase with inflammations, infections, or tissue damage. It is used as a monitoring marker in the acute phase of these pathologies. CRP has recently been investigated as a research marker of cardiovascular diseases, and recent studies have reported that increased CRP levels are associated with DM [86,121]. Zhu et al. observed higher plasma CRP levels in patients with PDN than in those with T2DM without neuropathy, consistent with previous findings reported by Doupis et al. [36,94]. From an immunological perspective, the increase in CRP could be a biomarker of immune responses mediated by IL-1, IL-6, and TNF-α [86].

### 5.3. Monocyte Chemoattractant Protein 1

MCP1 is a monomeric polypeptide belonging to the chemokine family. It is secreted by monocytes, macrophages, and dendritic cells from inflammatory foci and has a chemotactic and additional monocyte activation role [122]. MCP1 secretion is induced by increased levels of pro-inflammatory cytokines such as IL-1β, TNF-α, IFN-γ, and platelet-derived growth factor (PDGF), contributing to the relocation and recruitment of monocytes/mononuclear phagocytes under hypoxic conditions toward inflammatory sites [52]. Serum and urinary MCP1 levels are increased in both the early and late stages of T2DM. Spinal astrocytes and TNF-α could activate c-Jun-N-terminal kinase (JNK), and the activated TNF- α/JNK pathway would transiently stimulate MCP1 [52,123]. Therefore, its increase could be considered a useful biomarker for diagnosing neuropathic pain. A study carried out in the United Arab Emirates, which included 102 patients with T2DM, evidenced higher levels of MCP-1 in patients with confirmed PDN compared to those without. This high level persisted even after adjusting for various confounding factors such as gender, age, BMI, and HbA1c, suggesting a strong association between MCP-1 and PDN [17]. Il-8 and MCP-1 belong to the chemokine family [124], and the same study demonstrated their parallel increase in PDN patients [17]. Also, the MCP-1 level increase is associated with other inflammatory cytokines such as IL-6 and TNF-α which indicate the existence of an inflammatory cascade in patients suffering from PDN, further pointing to MCP-1′s involvement in these inflammatory pathways [17]. TGF-β is known for its anti-inflammatory and immunosuppressive properties, and interestingly, TGF-β levels significantly decreased in PDN patients compared to those that did not present PDN. The inverse relationship with MCP-1 suggests that the reduction in TGF-β is associated with the increase in the inflammatory response and the balance between MCP-1 and TGF-β could modulate the inflammatory response in PDN [17].

MCP-1 and its receptor, CCR2, play a role in the development of neuropathic pain, and MCP-1/CCR2 signaling is associated with the chronic pain mechanism of hypersensitivity [125]. By driving monocyte recruitment, monocyte infiltration, and inflammation, in conjunction with obesity and other inflammatory factors amplifying its effects, it may be a key factor contributing to nerve damage in diabetic neuropathy.

Current studies suggest that MCP-1 appears to be an important inflammatory biomarker involved in the inflammatory pathways associated with the development of PDN. We consider that further research is necessary for the full understanding of its role in PDN pathophysiology and its potential as a therapeutic target.

## 6. Vitamin D Deficiency and PDN

Research suggests that vitamin D plays a crucial role in regulating the balance between inflammation and immunosuppression [55]. Observational studies have revealed a correlation between vitamin D deficiency and heightened inflammation associated with conditions such as osteoarthritis [56], hypertension [126], hypertension during pregnancy [127], and foot-superimposed infections in patients with DM [128].

Recent studies have highlighted the anti-inflammatory properties of vitamin D. A lower level of 25-hydroxyvitamin D (25(OH)D) is associated with increased insulin resistance such as in metabolic syndrome, diabetes, and obesity, suggesting that its deficiency is involved in the appearance of insulin resistance. Despite the data provided by clinical research, there is a need to expand investigations and conduct well-designed randomized clinical trials to elucidate the molecular mechanisms [129].

Vitamin D status is classically determined by measuring the circulating level of calcidiol or 25(OH)D. The forms of vitamin D are represented by ergocalciferol (D2) and cholecalciferol (D3), and calcidiol and calcitriol (1,25-dihydroxy vitamin D3) are primary metabolites [129].

**Figure 6:**
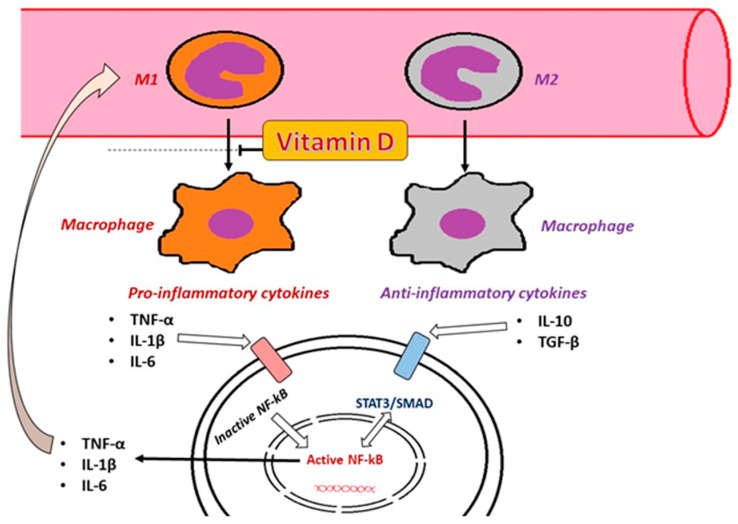
The role of monocytes (M1 and M2) in inflammation and the theoretical protective effect of vitamin D. The activation of M1-type monocytes will lead to pro-inflammatory macrophages in the interstitial tissue, affecting the cellular metabolism secondary to the secretion of pro-inflammatory cytokines TNF-α, IL-1β, and IL-6, leading to the activation of NF-κB. On the other hand, M2 monocytes, by developing in tissue macrophages, will secrete anti-inflammatory cytokines (IL-10), acting on two inhibitory intracellular proteins, such as signal transducer and activator of transcription 3 (STAT3) and signal transducer for receptors of the TGF-β family (SMAD), interfering in the activation of NF-κB. Studies showed an inhibitory effect of vitamin D in the conversion of M1 to pro-inflammatory macrophages, but without an impact on M2 macrophages.

Calcitriol regulates and improves pancreatic β cell function in T2DM through calcium-regulating receptors [130]. Other effects include increasing insulin sensitivity by stimulating the expression of its receptors, reducing chronic inflammation by deactivating inflammatory cytokines associated with increased insulin resistance, and protecting against apoptosis [131,132,133] (Figure 6).

The deficit of nerve growth factor (NGF) can lead to the development of small fiber neuropathy [72]. The secretion of NGF is promoted by vitamin D. Recent studies have highlighted positive correlations between vitamin D and NGF in T1DM [134,135]. In the presence of glucotoxicity, vitamin D can be deactivated by the CYP24A1 enzyme in Schwann cells, leading to reduced NGF secretion and favoring small nerve fiber neuropathy [136]. Numerous studies have shown a link between vitamin D deficiency and painful PDN. For example, Shillo et al. discovered that individuals with painful PDN had lower vitamin D levels than those with painless diabetic neuropathy or with T2DM without diabetic neuropathy [137]. Additionally, a single intramuscular dose of 600,000 IU of vitamin D3 effectively treated PDN and improved pain scores [138,139]. Increased inflammation in chronic conditions has been linked to vitamin D deficiency. Xiaohua et al. observed that among patients with T2DM, severe vitamin D deficiency (<10 ng/mL) was more common in those with painful PDN (25.8%) than in those without diabetic neuropathy (8.1%) or with painless PDN (12.5%; *p* < 0.01) [120]. Furthermore, the multivariate logistic analysis indicated a negative correlation between severe vitamin D deficiency and levels of IL-6 (r = −0.56, *p* < 0.01) and TNF-α (r = −0.47, *p* < 0.01) [120].

Tiwari et al. revealed a notable positive correlation between severe vitamin D deficiency and elevated levels of cytokines IL-1β (r = −0.323, *p* ≤ 0.001) and IL-6 (r = −0.154, *p* ≤ 0.040) among 112 patients with DM with foot infections and 109 diabetic controls [139]. Karonova et al. randomized 67 patients with T2DM and PDN into two treatment groups: low- (5000 UI) and high-dose (40,000 UI) cholecalciferol orally once weekly for 24 weeks. Of the 62 who completed the study, a significant percentage (78%) showed vitamin D deficiency at the initial screening, and neuropathy severity decreased in the high-dose group. A decrease in the level of IL-6 (2.5 vs. 0.6 pg/mL, *p* < 0.001) and an increase in the level of IL-10 (2.5 vs. 4.5 pg/mL, *p* < 0.001) was also observed in the high-dose group after 24 weeks. In addition, body max index (*p* = 0.001) and HbA1c levels (*p* = 0.004) decreased significantly in the high-dose group [140]. These studies suggest that a low level of vitamin D correlates with an increased level of pro-inflammatory cytokines, and vice versa, the correction of the deficiency can be followed by the increase in the level of anti-inflammatory cytokines with the reduction in inflammatory aggression on the peripheral nerve. It has been suggested that prophylactic doses of vitamin D should be administered to patients with obesity, with vitamin D supplementation reported to be associated with weight reduction, improved insulin resistance, and better HbA1c levels [141].

The REGARDS (Reasons for Geographic and Racial Difference in Stroke) study showed an association between a low serum 25(OH)D level and increased levels of IL-6 and CRP, without significant effects on IL-10 levels [142]. A meta-analysis of 24 randomized clinical trials reported lower levels of CRP and TNF-α in patients taking vitamin D supplements than in those not taking vitamin D supplements but did not report differences in IL-6 levels [143]. Other studies have shown that active forms of vitamin D reduce the levels of TNF-α and IL-6 and stimulate IL-10 production in immune cells [144].

Vitamin D is thought to influence the levels of inflammatory cytokines by activating NF-κB and modulating nuclear transcription factors involved in cytokine production (Figure 3). This effect is believed to occur through the regulation of genes encoding inflammatory cytokines and the interaction of vitamin D with specific elements in the promoters of these genes. Additionally, calcitriol regulates the production of inflammatory factors in macrophages through a calcium-dependent mechanism [145].

**Figure 7:**
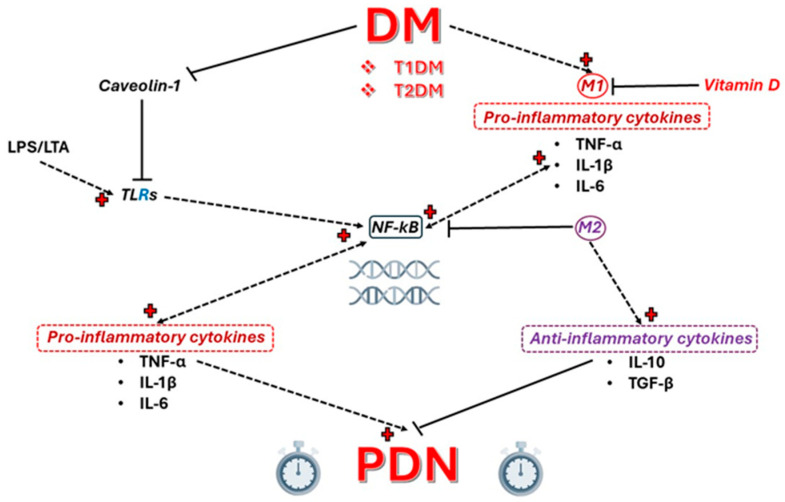
Schematic illustration presenting the development of PDN from DM. The inhibitory effects of DM (either T1DM or T2DM) decrease the levels of caveolin-1 with the concomitant activation of M1 monocytes, leading to pro-inflammatory effects, by the activation of NF-κB. The concentration of pro-inflammatory cytokines (TNF-α, IL-1β, and IL-6) seems to be lowered by vitamin D administration, left in the anti-inflammatory pathway of M2 monocytes, slowing down the progression of DM in PDN.

A summary of the effects of the pro-inflammatory and anti-inflammatory pathways, together with the evolution of DM in PDN, is described in Figure 7.

## 7. Potential Treatment Alternatives

One of the substances that was investigated for its anti-inflammatory and analgesic potential is 6-hydroxyflavanone (6-HF), which targets COX-2, 5-lipoxygenase, and GABA_A_ opioid receptors, with these effects being confirmed through in vitro and in vivo tests in rodent models. The authors demonstrated a significant inhibition, especially of 5-lipoxygenase and COX-2, resulting in a reduction in the inflammation associated with DM and the improvement of nociception in neuropathy induced by DM in animal studies [146].

Jia et al. found that the subcutaneous administration of 0.3 mg/kg daidzein (a CAV1 inhibitor) for 14 days attenuated the upregulation in CAV1 expression, improving pain hypersensitivity, presumably due to the reduction in TLR4 expression in the spinal cords of rats [45].

Li et al. demonstrated that fish oil supplementation inhibited NF-κB activation in diabetic rats by blocking the entry of NF-κB subunits into the nucleus, reducing its levels in the nucleus and maintaining it in the cytoplasm in its inactive form. This effect was associated with reduced inflammatory cytokine levels and an anti-apoptotic effect. NF-κB activation seems to play an important role in the early stages of painful PDN, and blocking it could be helpful before this complication develops [6].

The administration of anti-TNF-α antibodies or TNF-α inhibitors reduced hypersensitivity secondary to nerve damage, and the direct injection of TNF-α into the sciatic nerve produced painful neuropathy [147,148]. The inhibition of TNF-α by a recombinant human TNF receptor–antibody fusion protein improved nerve conduction velocity and reduced nerve fiber demyelination and axonal degeneration [149].

Infliximab is a monoclonal antibody targeting soluble and transmembrane TNF-α used in the treatment of various autoimmune disorders to reduce the biological activity of TNF-α. Kiortsis et al. performed a study that demonstrated the beneficial effects of infliximab treatment on insulin sensitivity in insulin-resistant patients with rheumatoid arthritis (RA) and ankylosing spondylitis (AS) [150]. Administering infliximab to diabetic mice resulted in a return to near-normal levels of sensory conduction velocity (SCV), motor conduction velocity (MCV), intraepidermal nerve fiber density, and phosphorylated NF-κB p65 staining in DRGs and improved thermal sensitivity after four weeks, approaching levels seen in patients without DM, although their levels did not normalize completely [22,151]. Furthermore, inhibiting TNF-α using a recombinant human TNF receptor–antibody fusion protein in STZ-induced diabetic rats improved the MCV and SCV, prevented abnormalities in myelin and peripheral nerve structure, and increased the expression of myelin basic protein (MBP) [22,149]. Thus, inhibiting pro-inflammatory cytokine TNF-α seems to be followed by an improvement in the pathological changes in the peripheral nerve, as was demonstrated in the aforementioned studies.

N-acetylcysteine (NAC) is a drug used mainly for its mucolytic properties. It has a good safety profile and provides antioxidant and anti-inflammatory effects by increasing the amount of glutathione (GSH) inside cells. In addition, it reduces the level of TNF-α, IL-1β, and IL-6 and suppresses NF-κB activity [4]. Administering N-acetylcysteine to diabetic rats, which inhibits TNF-α and acts as a free radical scavenger, among other functions, had beneficial effects on PDN, including the increased mean size of myelinated fibers compared to untreated rats [22,152]. Other substances, such as troglitazone and gliclazide, also showed comparable effects on myelinated fiber morphology after treatment [153].

Melatonin has been shown to inhibit NF-κB activation and neuroinflammation. Melatonin treatment reduced TNF-α, IL-6, COX2, and iNOS levels in sciatic nerves followed by improved microcirculation in the peripheral nerves of diabetic rats, contributing to improved motor nerve conduction [14,154].

The IL-1 antagonist anakinra reduced systemic inflammation in both T2DM and T1DM by improving blood glucose levels and protecting β cells. Administering the anti-inflammatory drug salsalate correlated with improved glycemic control in T2DM, as it is known that some pro-inflammatory cytokines such as TNF-α and IL-1β reduce insulin sensitivity and TNF-α, IL-1β, and IFN-γ can disturb intracellular calcium in β cells, preventing insulin release [90]. Administering salsalate, which targets the NF-κB pathway, to patients with T2DM had beneficial effects on CRP, triglycerides, and free fatty acid levels [155].

A separate study observed a significant decrease in the severity of neuropathy and an increase in IL-10 levels in patients with T2DM and PDN after treatment with cholecalciferol (40,000 IU/week) for 24 weeks [90].

The systemic or spinal administration of flucytosine, which is a non-selective metabolic inhibitor of glial cells, as well as minocycline, a selective inhibitor of microglia, reduced the production of IL-1β and TNF-α in these cells and led to a decreased level in rats, ameliorating thermal and mechanical hypersensitivity [84,85].

Inhibiting MCP1 activity could be used in the management of inflammation. A controlled trial on managing diabetic neuropathic pain found that administering deep tissue laser therapy twice weekly for four weeks, followed by once weekly for a further eight weeks, significantly decreased pain, which was accompanied by reductions in serum IL-6 and MCP1 levels. These data were consistent with other studies and suggest that MCP1 may be a promising therapeutic target for treating diabetic neuropathy [156,157].

The treatments currently administered in PDN mainly target neuropathic pain or represent preventive therapies. However, there are also promising recent advances aimed at accelerating therapeutic development. The most important classes of drugs used in PDN include antiepileptics, tricyclic antidepressants, and serotonin and norepinephrine reuptake inhibitors.

Small studies have shown that transcutaneous electrical nerve stimulation, pulse-dose electrical stimulation, and frequency-modulated electromagnetic neural stimulation were associated with increased tactile perception and pain reduction, but the underlaying mechanisms require further investigations. Other treatments, such as acupuncture and pulsed electromagnetic field therapy, have shown only temporary and inconclusive outcomes in painful diabetic neuropathy [158].

New studies conducted on significant patient cohorts are required to address the pathophysiological mechanisms involved in the onset of PDN.

## 8. Conclusions

Inflammatory syndrome seems to appear early in T1DM and especially T2DM, even from the IGT stage, and the increased incidence of PDN in T2DM suggests a possible causative effect. However, the role of inflammation in the etiopathogenesis of PDN requires additional clarification to facilitate lifestyle changes and encourage early interventions to prevent the onset of complications. Various factors, including vitamin D, can influence the levels of inflammatory cytokines. Since vitamin D deficiency is a potentially modifiable risk factor for PDN, its correction could reduce the prevalence of PDN.

The phenotype of monocytes seemed to be altered under hyperglycemic conditions, and this alteration can persist after differentiation in macrophages, even after glycemic corrections. Reducing macrophage recruitment or interfering with their polarization, favoring an anti-inflammatory M2 phenotype, could prevent or ameliorate DM complications in the future.

Research into various substances and treatments for painful diabetic neuropathy has revealed multiple promising approaches, including anti-inflammatory agents, TNF-α inhibitors, antioxidants, and novel therapies. New studies with larger patient cohorts are needed to address the pathophysiological mechanisms involving inflammation underlying PDN for a better understanding and to confirm the treatment’s effectiveness.

## Data Availability

Data are available based on the request from corresponding author.

## Abbreviations

ADA	The American Diabetes Association
ADIPOQ	Adiponectin C1Q
AGEs	Advanced glycation end products
ATP	Adenosine triphosphate
AS	Ankylosing spondylitis
CAV1	Caveolin 1
CD	Cluster of differentiation
CNS	Central nervous system
COX2	Cyclooxygenase 2
CoQ10	Coenzyme Q10
CRP	C reactive protein
CXCL10	C-X-C motif chemokine ligand 10
Cyt C	Cytochrome C
DAN	Diabetic autonomic neuropathy
DM	Diabetes mellitus
DNA	Deoxyribonucleic acid
DRG	Dorsal root ganglion
25(OH)D	25-hydroxyvitamin D
GSH	Glutathione
HbA1c	Hemoglobin A1c
ICAM1	Intercellular adhesion molecule 1
IFN	Interferon
IGT	Impaired glucose tolerance
IκB	Inhibitor of kappa B
IL	Interleukin
IL-R	Interleukin receptor
iNOS	Inducible nitric oxide
IRS	Insulin receptor substrate
JAK	Janus kinase
JNK	Jun-N-terminal kinase
LEP	Leptin
LPS	Lipopolysaccharide
LTA	Lipoteichoic acid
M1	Monocyte type 1
M2	Monocyte type 2
MBP	Myelin basic protein
MCP1	Monocyte chemoattractant protein 1
MCV	Motor conduction velocity
MMP12	Matrix metallopeptidase 12
mRNA	Messenger ribonucleic acid
MS4A1	Membrane-spanning 4-domains A1
NGF	Nerve growth factor
NAC	N-acetylcysteine
NF-κB	Nuclear factor kappa B
NK	Natural killer
NO	Nitric oxide
Nrf2	Nuclear factor-2 erythroid-related factor-2
PDGF	Platelet-derived grown factor
PDN	Peripheral diabetic neuropathy
RA	Rheumatoid arthritis
REGARDS	Reasons for Geographic and Racial Difference in Stroke
rER	Rough endoplasmic reticulum
SCV	Sensory conduction velocity
SMAD	Signal transducer for receptors of the transforming growth factor beta family
STAT3	Signal transducer and activator of transcription 3
STZ	Streptozocin
T1DM	Type 1 diabetes mellitus
T2DM	Type 2 diabetes mellitus
Th1	T helper 1
TLR	Tool-like receptor
TNF-R	Tumor necrosis factor receptor
TNF-α	Tumor necrosis factor alpha
TNFRSF1A	TNF receptor superfamily member 1A
TNFRSF1B	TNF receptor superfamily member 1B
TNF-β	Tumor necrosis factor beta
TGF-β	Transforming growth factor-β
